# Effects of breed and early feeding on intestinal microbiota, inflammation markers, and behavior of broiler chickens

**DOI:** 10.3389/fvets.2024.1492274

**Published:** 2024-12-02

**Authors:** Francesca Marcato, Dirkjan Schokker, Soumya Kanti Kar, Alex Bossers, Frank Harders, Johanna M. J. Rebel, Christine A. Jansen, Elianne van der Valk, Leo Kruijt, Dennis Elbert te Beest, Ingrid C. de Jong

**Affiliations:** ^1^Wageningen Livestock Research, Wageningen University & Research, Wageningen, Netherlands; ^2^Wageningen Bioveterinary Research, Wageningen University & Research, Lelystad, Netherlands; ^3^Institute for Risk Assessment Sciences, Utrecht University, Utrecht, Netherlands; ^4^Cell Biology and Immunology Group, Wageningen University & Research, Wageningen, Netherlands; ^5^Biometris, Wageningen University & Research, Wageningen, Netherlands

**Keywords:** broilers, breed, early feeding, gut microbiome, immunity, behavior

## Abstract

Recently, the Netherlands has shifted toward more welfare-friendly broiler production systems using slower-growing broiler breeds. Early post-hatch feeding (EF) is a dietary strategy that is currently used in commercial broiler production to modulate the gut microbiota and improve performance and welfare. However, there is a knowledge gap in how both breed and EF and their interplay affect gut microbiota composition and diversity, inflammatory status, and broiler behavior. Therefore, the aim of this study was to investigate the effects of breed (fast vs. slower-growing), EF, and their interaction on jejunum microbiota, inflammation, and behavior of broiler chickens. The study included a total of 416 Ross 308 and 416 Hubbard JA757 day-old male broiler chickens, observed until they were 37 days and 51 days old, respectively. Within each breed, one-half of the chickens received EF and the other half did not. A total of two chickens per pen were euthanized at two time points, that is, target body weight (BW) of 200 g and 2.5 kg, and jejunum samples were collected. The jejunum content samples (*N* = 96) were analyzed for their microbiota, whereas the jejunum tissue (*N* = 96) was used for the detection of mRNA levels of cytokines (IL-17, IL-22, and IFNγ). Two behavioral tests were performed to assess fear responses: (1) a novel environment test at a target BW of 200 g and (2) a tonic immobility test at a target BW of 2.5 kg. Breed affected the microbiota at a target BW of 2.5 kg (*p* = 0.04). A breed × EF interaction (*p* = 0.02) was present for IFNγ at a target BW of 200 g. During the novel environment test, Ross 308 chickens exhibited a shorter latency to vocalize and a higher number of vocalizations compared to Hubbard JA757 chickens (*p* < 0.05). Early-fed broiler chickens vocalized less compared to not early-fed chickens (Δ = −27.8 on average; *p* < 0.01). During the tonic immobility test, Hubbard JA757 chickens exhibited a shorter latency to stand compared to Ross 308 chickens. In conclusion, using a slower-growing breed has beneficial effects on gut microbiota and fear responses of broilers, especially at slaughter age, whereas EF seems to have an impact only at an early stage of the life of broilers.

## 1 Introduction

In broiler chickens, as in other species, gut health is essential for feed efficiency, growth, and health given the relationship with nutrient utilization, macro- and micro-structural integrity of the gut, the stability of the microbiota, and inflammatory status (1–4). A compromised gut health can affect digestion and nutrient absorption, which in turn may have a detrimental effect on feed efficiency and lead to greater susceptibility to diseases, leading to economic losses (4). Many factors can affect gut health, such as age, sex, breed, diet, and litter conditions (5, 6). In particular, breed and nutritional strategies, such as early post-hatch feeding, are the ones playing a major role in modern broiler production (7–11). With regard to breed, the strong selection in broilers toward traits such as high feed intake and rapid growth has shown to have adverse effects on the animals such as metabolic disorders (i.e., ascites syndrome), a low responsiveness of the immune system coupled with low-grade chronic inflammation, and a decreased resistance to pathogens (12–15). In the Netherlands, there has been a shift toward new broiler production systems using slow-growing broiler breeds housed at reduced stocking densities (25 kg/m^2^) compared to the conventional production system using fast-growing broiler breeds with a stocking density of 38 kg/m^2^ or higher (16–18). Previous research (9) showed that there were differences between slower-growing (Hubbard JA757) and fast-growing broilers (Ross 308) in performance, fecal endotoxin release, and a tendency for a different fecal microbiota at slaughter weight. Ross 308 chickens exhibited better performance, as expected, but also higher fecal endotoxin levels and higher alpha diversity index of fecal microbiota at slaughter weight than Hubbard JA757 chickens (9). However, fecal samples might not be entirely representative of the whole gut microbiota, and jejunum might provide a better understanding of the effects of host genotype on gut microbiota composition. In addition, that study (9) did not include any data on markers of gut inflammation and behavior of these two lines of broilers at both early (at a target BW of approximately 200 g) and late age (at a target BW of approximately 2.5 kg), which together could serve as a basis to better understand and improve welfare in these animals.

Another factor known to affect gut health is the early provision of feed and water directly after hatch, also known as early post-hatch feeding (EF) (8). The period from late chick embryonic development to the first few days following hatching is a critical period for the development of the gastrointestinal tract and immune system in poultry (19), and during the first days post-hatch, the interplay between nutrition and microbiota regulates intestinal epithelial cell composition and homeostasis (20, 21). Studies have shown positive effects of EF on growth, nutrient utilization, gut integrity, immunity, and fear responses (7, 22–25) compared to delayed feeding. However, there is still a debate about whether the effects of EF strategy can affect inflammatory markers in the gut and behavior in both the short and long term. Moreover, the effects of EF were mainly assessed in fast growers, indicating that a better insight into the effects on slower growers is needed.

Given the aforementioned background, the current study aimed to investigate the effects of both breed and EF and their interaction on jejunum microbiota, immune development, and behavior at two different ages (approximately a target BW of 200 g and 2.5 kg, respectively). The hypothesis was that slower-growing broiler chickens and early-fed chickens had a higher amount of microbial species, had fewer signs of gut inflammation, and were less fearful compared to faster-growing and not early-fed chickens.

## 2 Materials and methods

### 2.1 Experimental design

The study was conducted at the experimental research facility of Wageningen University and Research and complied with the Dutch law on animal experiments. The project was approved by the Central Commission on Animal Experiments (license number AVD4010020197985; experiment no. 2019.D-0009.002), and the experiment was approved by the Ethics Committee of Wageningen University and Research, the Netherlands. The experiment was set up as a complete 2 × 2 × 2 factorial arrangement with three factors: (1) breed (fast-growing Ross 308 vs. slower-growing Hubbard JA757); (2) no use vs. combined use of probiotics and prebiotics in the diet and the drinking water; (3) EF at the hatchery vs. non-early feeding. The complete experimental design and effects of all factors are described in a previous study (9). The current study will focus only on the effects of EF and breed, excluding the effects of prebiotics and probiotics. Thus, the treatment groups in the current study were the following:

R + EF = Ross 308 broilers with early feeding.

R - EF = Ross 308 broilers without early feeding.

H + EF = Hubbard JA757 broilers with early feeding.

H - EF = Hubbard JA757 broilers without early feeding.

A power analysis was conducted before the start of the experiment, and it was based on one of the indicators (endotoxin concentration) described in the previous companion study (9). From these calculations, it was estimated that six replicates per treatment group would be sufficient for the study. This experiment included a total of 416 fast-growing (Ross 308; breeder age of 45 weeks) and 416 slower-growing (Hubbard JA757; breeder age of 45 weeks) day-old male broiler chickens obtained from a commercial hatchery (Probroed & Sloot, Lunteren, the Netherlands). Upon arrival at the research facility, chicks were neck-tagged for individual identification and randomly allocated to their respective pen. Each pen measured 1.10 × 1.90 m (L × W). Two identical climate-controlled rooms, each containing 24 pens, were used to house the chickens, of which 12 pens per room were included in the present trial (thus 24 pens in total). The broilers were allocated to the pens (26 chickens/pen) according to a completely randomized block design, which consisted of six blocks of eight pens equally distributed in the two rooms. A companion study (9) includes all the other details on the experimental design of this study.

### 2.2 Feeding program and treatments

A three-phase feeding program was applied, and all treatment groups received an identical diet formulated by ForFarmers (Lochem, the Netherlands) and produced by Research Diet Services B.V. (Wijk bij Duurstede, the Netherlands). The diet was formulated in such a way that it was intermediate to the guidelines for both breeds. A starter diet was provided between days 0 and 14 (ME = 2,934 kcal/kg; CP = 218.2 g/kg; dLys = 12.4 g/kg), a grower diet between days 14 and 37 (ME = 3,023 kcal/kg; CP = 190.3 g/kg; dLys = 10.1 g/kg), and a finisher diet (only provided to Hubbard JA757) between days 37 and 51 (ME = 3,075 kcal/kg; CP = 184.5 g/kg; dLys = 9.6 g/kg). Chickens belonging to treatment group R + EF and H + EF also received a prestarter diet at the hatchery (ME = 3,048 kcal/kg; CP = 211.9 g/kg; dLys = 12.4 g/kg). The full composition of all diets is described in the companion study (9).

### 2.3 Sampling moments and measurements

#### 2.3.1 Jejunum microbiota

A total of 96 chickens (24 chickens/treatment group) were selected for several individual measurements at two sampling moments. At an early stage with a target BW of approximately 200 g (day 8 for Ross 308 and day 9 for Hubbard JA757 chickens), two chickens/pen (48 chickens in total) were euthanized by cervical dislocation and dissected. At a late stage with a target BW of approximately 2.5 kg (day 34 for Ross 308 and day 50 for Hubbard JA757 chickens), two animals/pen (48 chickens in total) were euthanized by electrocution and dissected. The H2H Euthanizer (H2H-230 V, Top Equipment B.V., Lienden, The Netherlands) was used to euthanize broiler chickens via electrocution. At each sampling moment, a total of 48 samples of jejunum content were collected into Eppendorf tubes and immediately freeze-dried in liquid nitrogen. Thereafter, the samples were stored at −80°C until the analyses of the jejunum microbiota composition and diversity, which were carried out at Wageningen Bioveterinary Research (Lelystad). The samples were used for DNA extraction with the Invitrogen PureLink Genomic DNA Mini Kit (Thermo Fisher Scientific, Waltham, MA), according to the manufacturer’s instructions. Total DNA was quantified using an Agilent 2,200 TapeStation (Santa Clara, United States). The hypervariable regions V3 and V4 of the 16S rRNA gene were amplified in a limited cycle PCR with the primers CVI_V3-forw CCTACGGGAGGCAGCAG and CVI_V4-rev GGACTACHVGGGTWTCT. The following amplification conditions were used as previously described (26): 98°C for 2 min, followed by 20 cycles of 98°C for 10 s, 55°C for 30 s, and 72°C for 10 s, and finally by 72°C for 7 min. PCR products were checked on TapeStation (Agilent, Santa Clara, CA) and after barcoding subsequently sequenced on a MiSeq sequencer (Illumina Inc., San Diego, CA) using a version 3 paired-end 300 bp kit. The amplicon sequences were first demultiplexed and subsequently filtered, trimmed, error-corrected, dereplicated, chimera-checked, and merged using the DADA2 package [v.1.16.0 (27)]. By using the standard parameters except for TruncLength = (270,220), trimLeft = (25,33), and minOverlap = 10, reads were classified against the SILVA v.138.1 database.

#### 2.3.2 Cytokines

A jejunum sample was collected from the same 96 animals dissected at a target BW of 200 g (day 8 for Ross 308 chickens and day 9 for Hubbard JA757 chickens) and 2.5 kg (day 34 for Ross 308 chickens and day 50 for Hubbard JA757 chickens) to quantify cytokine gene expression. Approximately 2 cm of intestine was collected, just after the Meckel’s diverticulum. The tissue was directly freeze-dried in liquid nitrogen and stored at −80°C until further processing. From each tissue sample, a piece of approximately 30 mg was collected and placed in an Eppendorf tube with QIAzol Lysis Reagent (Qiagen, Cat. No. ID:79306) and a 5-mm stainless steel bead. The tissue was then lysed using a TissueLyser II (Qiagen) two times for 3 min at 20 Hz, and the RNA was extracted using the RNeasy Plus Universal Mini Kit (Qiagen, Cat. No. ID: 73404). The RNA concentration was checked using the NanoDrop, and RNA quality was checked using the Bioanalyzer (Bioanalyzer kit: Agilent RNA 6000 Nano Kit Part Number 5067–1511). The RNA was synthesized into cDNA using reverse transcriptase according to the manufacturer’s instructions (Superscript III, Thermo Fisher, 18,080,093).

The RNA was synthesized into cDNA using reverse transcriptase (Superscript III, Thermo Fisher, 18,080,093). Then, 500 ng of RNA was mixed with random primers (250 ng) and dNTP mix (final concentration 1,66 mM) and was incubated for 5 min at 65°C followed by a cooling step of 4°C for at least 2 min. Afterward, DTT (final concentration 5 nM), first strand buffer, and Superscript III were added to the mixture. This mixture was then incubated according to the manufacturer’s protocol (5 min 25°C, 60 min 50°C, 15 min 55°C, 15 min 70°C, and infinite 4°C).

The cDNA of each jejunum sample was diluted 50x and amplified after a preincubation of 20 s at 95°C, followed by 40 cycles (1 s at 95°C and 20 s at 60°C) on a Fast Real-Time PCR System (Quantstudio5, Applied Biosystems) using the Sensifast SYBR Lo-Rox (Meridian Bioscience, BIO-94020) and primer mix (final concentration of 200 nM each). The primer sequences are shown in Supplementary Table 1. These primers are all intron-spanning and have a primer efficiency of 90–100%. The melting curves confirmed that a single amplicon was produced for each primer set.

qPCR analyses were performed by employing the double delta Ct (ΔΔCt) method. With this method, the gene expression of a total of three housekeeping genes (ACTB, PPIA, and RPRLPO) was compared to three genes of interest (IL-17, IFNγ, and IL-22). The most two reliable housekeeping genes (ACTB and PPIA) were then retained for subsequent analyses. For both housekeeping genes and the genes of interest, the control samples were represented by the average values of all animals belonging to the same treatment group (R + EF, R-EF, H + EF, or H-EF) at either an early or late sampling moment.

#### 2.3.3 Immune cells

At a target BW of approximately 2.5 kg (day 35 for Ross 308 chickens and day 49 for Hubbard JA757 chickens), 10 mL of blood was collected by venipuncture of the wing vein in 3 K-EDTA tubes (96 chickens in total), to determine absolute counts of several lymphocyte subsets in whole blood (28) using BD Trucount™ Tubes (BD Biosciences, San Jose, USA), according to the manufacturer’s instructions. The antibody mix consisted of the pan leukocyte marker mouse-anti-chicken-CD45-PE, the T-cell recognizing antibodies mouse-anti-chicken-CD3-PB and mouse-anti-chicken-CD4-APC, and the B-cell recognizing antibody mouse-anti-chicken-BU-1-FITC. All antibodies were obtained from SouthernBiotech (Birmingham, USA). Samples were measured using the Cytoflex LX Flow Cytometer (Beckman), and approximately 10,000 beads were recorded per sample. An analysis was performed using the software program FlowJo 10.10.0 (Tree Star Inc., Ashland, OR, USA), and the absolute cell counts were calculated.

#### 2.3.4 Behavior

##### 2.3.4.1 Novel environment test

On day 6 of age, two chickens per pen (both Ross 308 and Hubbard JA757) were selected for the novel environment test (NE) to measure separation anxiety by using a similar protocol to De Haas et al. (29). The test was conducted outside the pen, and a large black bucket (40 cm diameter × 50 cm height) was used to prevent chicks from escaping. All the chicks were placed into the bucket, and, during the test, the observer was out of sight of the test subjects. The behavioral response was recorded individually for 2 min, which included the number of vocalizations and escape attempts as well as the latency (in seconds) to the first vocalization and escape attempt.

##### 2.3.4.2 Tonic immobility test

The tonic immobility (TI) test was performed on approximately 2.5 kg target BW, thus on day 34 of age for Ross 308 chickens and on day 48 of age for Hubbard JA757 chickens, to test for fearfulness and cognitive development. The test was conducted following the procedure of Hollemans et al. (23) and modified by Giersberg et al. (30). Chickens were manually restrained on their back in a metal cradle, and direct contact with their eyes was avoided. After 10 s from restraining, the latency from immobility until the bird’s first attempt to erect itself (i.e., first leg, wing, or rump movement) was recorded. If the latency was >300 s, the test was ended and a maximum latency of 300 s was noted. The number of vocalizations and attempts to return to a standing position were also recorded.

### 2.4 Statistical analyses

Statistical analyses for the microbiota data were performed within the R environment (v4.3.2). The Phyloseq (v1.46.0) package was used for processing and statistical analyses and ggplot2 (v3.4.4) for visualization. The samples (*n* = 96) were pruned, where the sample sums needed to be equal to or higher than 10,000; this resulted in four samples being removed for downstream analyses. Thereafter, the samples were rarified to even depth (i.e., resample an OTU/ASV table, such that all samples have the same library size), resulting in a sample sum of 12,887 comprising 1,057 taxa. For alpha diversity measures, we focused on species richness, Shannon index, and Pielou’s evenness, whereas for beta diversity measures, the methods redundancy analysis (RDA) and principal coordinate analysis (PCoA) were used, and for distance, the Bray–Curtis dissimilarity was used. To assess the microbiota composition, the focus was on the top 10 taxa at a certain taxonomic level, such as phylum or genus, while all remaining taxa were aggregated into a single group labeled “Other.”

The other statistical analyses were carried out in SAS version 9.4 (SAS Institute Inc.). Residuals were always checked for normality and homogeneity of variance, and the variables were log-transformed when needed. For all analyses, the following model was used:


$$Y_{ijk}=\mu +{\mathrm{Breed}}_{i}+{\mathrm{Early feeding}}_{k}+\left({\mathrm{Breed}}_{i}\times {\mathrm{Early feeding}}_{k}\right)+\varepsilon_{ik}$$

where

- Y_ik_: response variable
- μ: overall mean
- Breed_i_: Ross 308 or Hubbard JA757 chickens
- Early feeding_k_: yes or no
- ε_ik_: residual error

Cytokines obtained from the jejunum samples were analyzed per time point and, together with the number of blood immune cells, were analyzed as continuous variables using a linear mixed model (LMM). Random pen nested in-room effects were also included in the model, and approximate *F*-tests (31) were used for fixed effects. Subsequent pairwise comparisons were performed using Fisher’s least significant difference method. In all analyses, effects with a *p*-value of ≤ 0.05 were considered significant. Immune cells expressed as % were analyzed using a generalized linear mixed model (GLIMMIX) comprising a logit link function and the Bernoulli variance as an “error” variance. Behaviors measured during the novel environment test including latency to first vocalization, latency to first escape attempt, and number of vocalizations were analyzed using an LMM. A number of escape attempts were categorized into four classes (0, 1, 2, and > 2) and analyzed using ordinal regression. With the exclusion of latency to the first escape attempt which was analyzed with an LMM, the other behaviors measured during the tonic immobility test required different analyses due to the non-normal distribution of data (>50% of data was 0). The number of attempts to stand and the number of vocalizations were both categorized into three classes (0, 1, and > 1) and analyzed using ordinal regression. Tonic immobility was categorized into three classes: (0 = 0 < TI < 100 s; 1 = 100 < TI < 300 s, and 2 = 300 s) and analyzed using ordinal regression.

## 3 Results

### 3.1 Effects on gut microbiota composition

**Figure 1:**
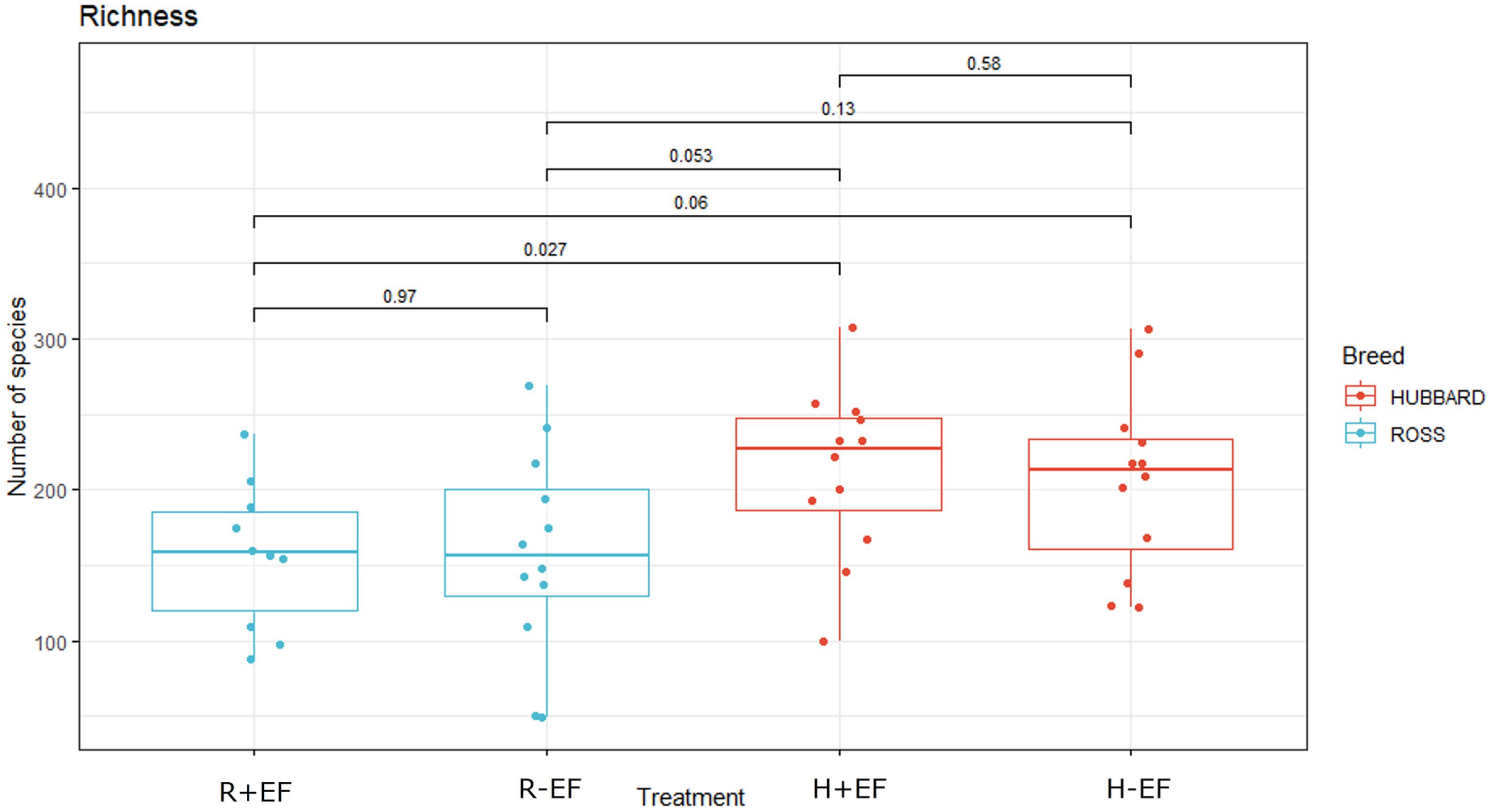
Effects of broiler breed (Ross 308 vs. Hubbard JA757) on observed species in samples of jejunum content collected at a late sampling moment (target BW ≈ 2.5 kg). R + EF = Ross 308 chickens with early feeding; R-EF = Ross 308 chickens without early feeding; H + EF=Hubbard JA757 chickens with early feeding; H-EF = Hubbard JA757 chickens without early feeding.

**Figure 2:**
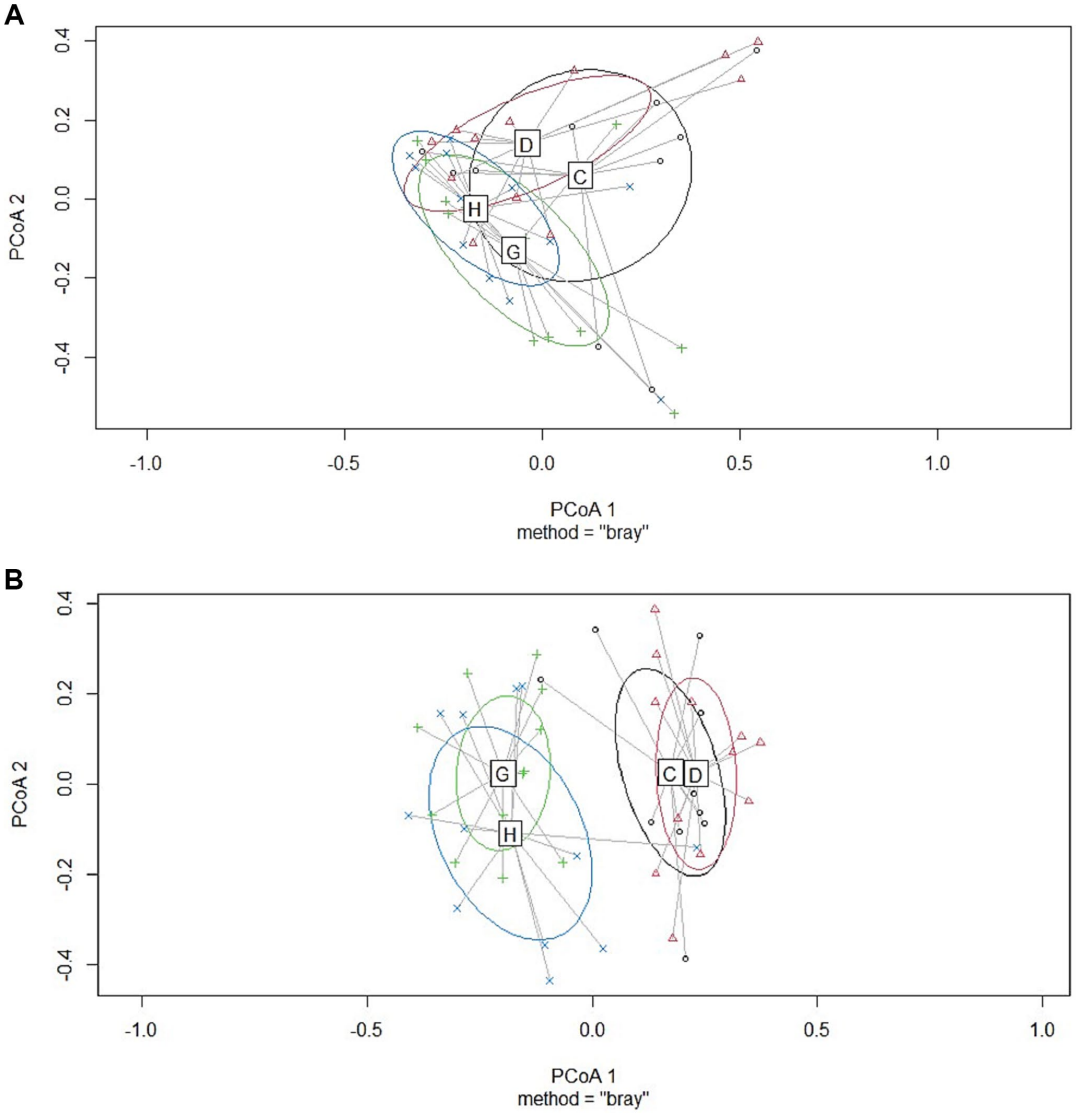
Results of principal coordinate analysis of jejunum samples collected from broiler chickens at two sampling moments [early, at a target BW ≈ 200 g; **(A)**; and late, at a target BW ≈ 2.5 kg; **(B)**]. C= Ross 308 chickens with early feeding; D= Ross 308 chickens without early feeding; G=Hubbard JA757 chickens with early feeding; H= Hubbard JA757 chickens without early feeding.

No significant differences were observed between the treatment groups for alpha diversity observed species and the Shannon index at the early sampling moment (target BW ≈ 200 g; see Table 1). At a late sampling moment, the broiler breed affected the observed species, with Ross 308 chickens having a lower number of observed species than Hubbard JA757 chickens (Table 1; Figure 1; *p* < 0.01). No effects were present for the Shannon index. The results of the principal coordinate analysis (PCoA) showed significant differences between broiler breed at both early (target BW ≈ 200 g; *p* < 0.01; Figure 2A) and late sampling moments (target BW ≈ 2.5 kg; *p* < 0.01; Figure 2B), whereas no significant effects were found for EF.

**Table 1:** Effects of broiler breed (Ross 308 vs. Hubbard JA757) and early feeding (EF; YES vs. NO) and their interaction on alpha diversity indices measured in samples of jejunum content at two sampling days.

	Treatments^1^					*p*-values		
	R + EF	R-EF	H + EF	H-EF	Pooled SD^2^	Broiler breed	EF	Broiler breed×EF
Early sampling day (target BW ≈ 200 g)								
Observed species	60	51	58	48	19	0.69	0.14	0.95
Shannon index	1.84	1.94	1.72	1.82	0.43	0.35	0.44	0.97
Late sampling day (target BW ≈ 2.5 kg)								
Observed species	157^a^	158^a^	213^b^	205^b^	58	<0.01	0.92	0.89
Shannon index	2.72	2.57	2.92	2.88	0.57	0.15	0.61	0.75

1 R + EF = Ross 308 chickens with early feeding; R-EF = Ross 308 chickens without early feeding; H + EF=Hubbard JA757 chickens with early feeding; H-EF = Hubbard JA757 chickens without early feeding.

2 SD, standard deviation.

Breed differences were further explored at the genus level, by comparing Ross and Hubbard chickens within the early-fed or non-early-fed treatments. Supplementary Figure 1 shows the composition at the genus level for both target BW ≈ 200 g and target BW ≈ 2.5 kg. Comparing single genera at a target BW ≈ 200 g for the top 10 most abundant genera showed only differences between the non-early-fed and early-fed treatments at a target BW ≈ 2.5 kg. Within the non-early-fed chickens, HT002, *Limosilactobacillus* and *Lactobacillus* were more present in Ross-EF vs. Hubbard-EF, while Hubbard-EF had more *Ligilactobacillus*, *Streptococcus,* and *Enterococcus* as compared to Ross-EF. Within the early-fed chickens (*p* < 0.05), Ross had a higher presence of HT002 and *Limosilactobacillus* in jejunum content, while Hubbard had more *Peptostreptococcaceae*, *Enterococcus*, and *Ligilactobacillus* (*p* < 0.001; Supplementary Figure 1; Supplementary Table 2).

### 3.2 Effects on cytokines

**Figure 3:**
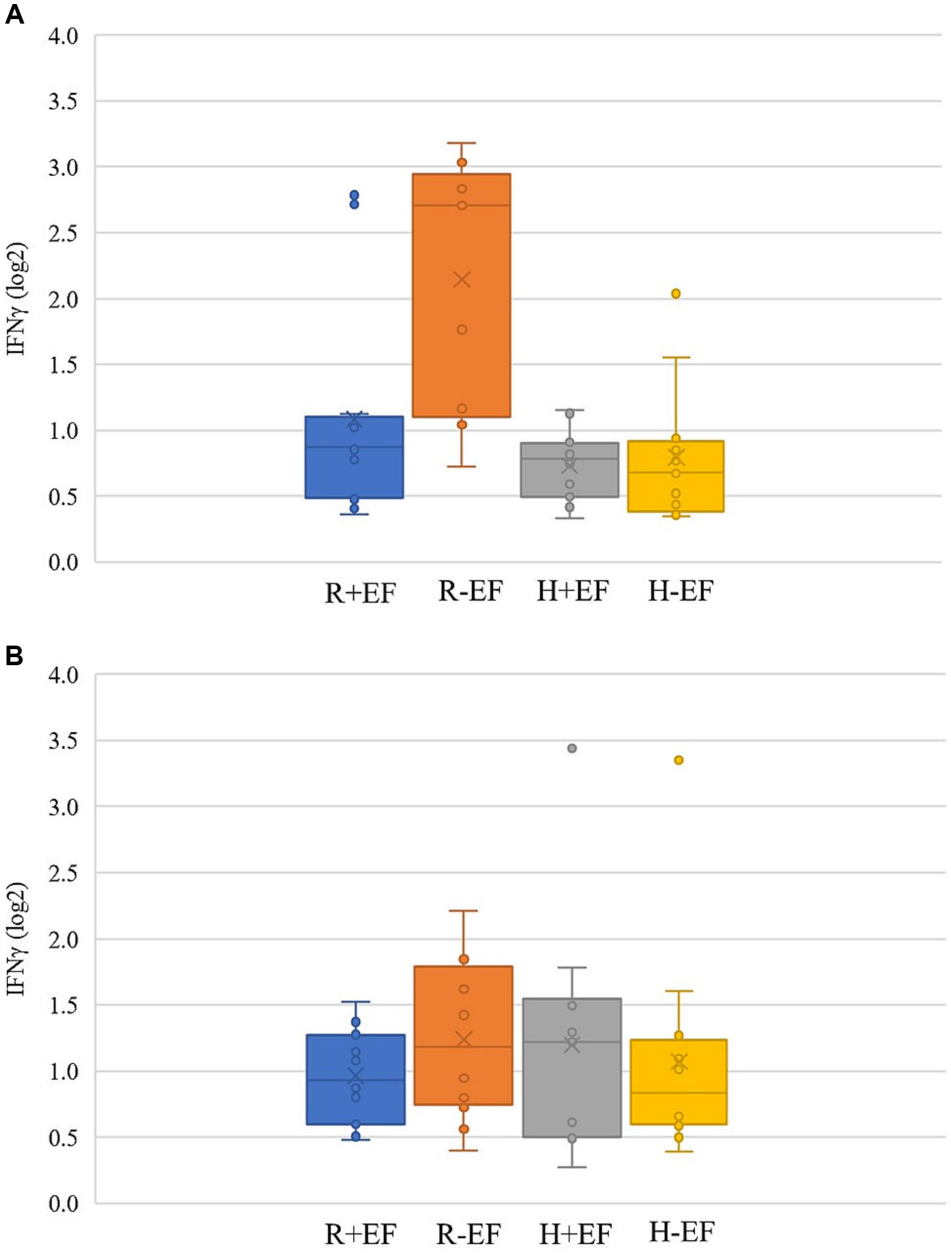
IFNγ expression level [(log2) fold change as compared to the treatment group average] in jejunum samples collected from broiler chickens at two sampling moments [early, at a target BW ≈ 200 g; **(A)**; and late, at a target BW ≈ 2.5 kg; **(B)**]. R + EF = Ross 308 chickens with early feeding; R-EF = Ross 308 chickens without early feeding; H + EF=Hubbard JA757 chickens with early feeding; H-EF = Hubbard JA757 chickens without early feeding.

The effects of treatments on cytokines measured in the jejunum of broiler chickens at two sampling moments are shown in Table 2 and Figures 3A,B. A significant interaction breed × EF was present at a target BW ≈ 200 g for expression of IL-17 and IFNγ (*p* ≤ 0.05; Table 2), whereas a tendency was present for IL-22 (*p* = 0.06; Table 2). In particular, broiler chickens without EF and from the fast-growing line Ross 308 showed higher IFNγ mRNA levels as compared to the other treatments (Figure 3A). No significant effects were found at a target BW ≈ 2.5 kg.

**Table 2:** Effects of broiler breed (Ross 308 vs. Hubbard JA757) and early feeding (EF; YES vs. NO) and their interaction on cytokine expression level (log2) fold change as compared to the treatment group average measured from jejunum samples collected from broiler chickens at two sampling moments.

	Treatments^1^					*p*-values		
	R + EF	R-EF	H + EF	H-EF	Pooled SD^2^	Broiler breed	EF	Broiler breed×EF
Early sampling day (target BW ≈ 200 g)								
IL-17	1.19	0.98	0.82	1.31	0.23	0.84	0.39	0.05
IL-22	1.25	0.91	0.92	1.12	0.21	0.68	0.68	0.06
IFNγ	1.08	2.14	0.73	0.80	0.25	<0.01	0.02	0.02
Late sampling day (target BW ≈ 2.5 kg)								
IL-17	1.61	1.83	1.81	1.36	0.80	0.57	0.46	0.63
IL-22	0.89	1.23	1.29	1.18	0.53	0.90	0.52	0.22
IFNγ	0.96	1.24	1.20	1.07	0.28	0.65	0.72	0.60

1 R + EF = Ross 308 chickens with early feeding; R-EF = Ross 308 chickens without early feeding; H + EF=Hubbard JA757 chickens with early feeding; H-EF = Hubbard JA757 chickens without early feeding.

2 SD, standard deviation.

### 3.3 Effects on immune cells

Broiler breed had a significant effect on the number of immune cells collected at a target BW ≈ 2.5 kg, with Hubbard JA757 chickens having a higher amount of leukocytes, T cells, and B cells in the blood than Ross 308 chickens (Table 3). The only significant interaction broiler breed × EF was present for B cells (Table 3), where a higher number of B cells were observed in Ross 308 chickens with early feeding compared to Ross 308 chickens without early feeding (Δ = 22.8 cells/μl) but to a lower amount of B cells (Δ = −36.7 cells/μl) for Hubbard JA757 chickens with early feeding compared to Hubbard JA757 chickens without early feeding. Broiler breed contributed also to significant differences in the percentage of T cells and CD4^+^ T cells (*p* < 0.05; Table 3), with Ross 308 chickens having a lower proportion of T cells and a higher proportion of CD4^+^ T cells than Hubbard JA757 chickens.

**Table 3:** Effects of broiler breed (Ross 308 and Hubbard JA 757) and early feeding (EF; YES or NO) and their interaction on immune cell parameters measured in the blood of male broilers collected on the dissection day (day 35 for Ross 308 and day 49 for Hubbard JA757).

	Treatments^1^					*p*-values		
	R + EF	R-EF	H + EF	H-EF	Pooled SD^2^	Broiler breed	EF	Broiler breed×EF
No. of cells/μl								
CD45	1199.3	1060.0	2015.9	2131.7	75.7	<0.01	0.88	0.13
B cells	51.8	29.0	159.7	196.4	12.7	<0.01	0.65	0.05
T cells	725.2	619.9	1549.2	1649.1	61.5	<0.01	0.95	0.14
CD4+ T cells	541.2	438.3	1025.8	1096.7	51.6	<0.01	0.74	0.13
CD4neg T cells	177.0	175.5	474.2	510.1	26.9	<0.01	0.84	0.83
%								
CD45	0.8	0.6	0.8	1.3	0.1	0.35	0.90	0.39
B cells	4.2	2.7	7.3	8.9	0.6	0.07	0.44	0.19
T cells	59.6	59.4	73.5	77.4	2.9	<0.01	0.57	0.40
CD4+ T cells	71.2	68.9	63.4	66.9	3.0	0.04	0.87	0.22
CD4neg T cells	27.7	30.3	28.9	30.5	2.7	0.48	0.31	0.63

1 R + EF = Ross 308 chickens with early feeding; R-EF = Ross 308 chickens without early feeding; H + EF=Hubbard JA757 chickens with early feeding; H-EF = Hubbard JA757 chickens without early feeding.

2 SD, standard deviation.

### 3.4 Effects on behavior

**Figure 4:**
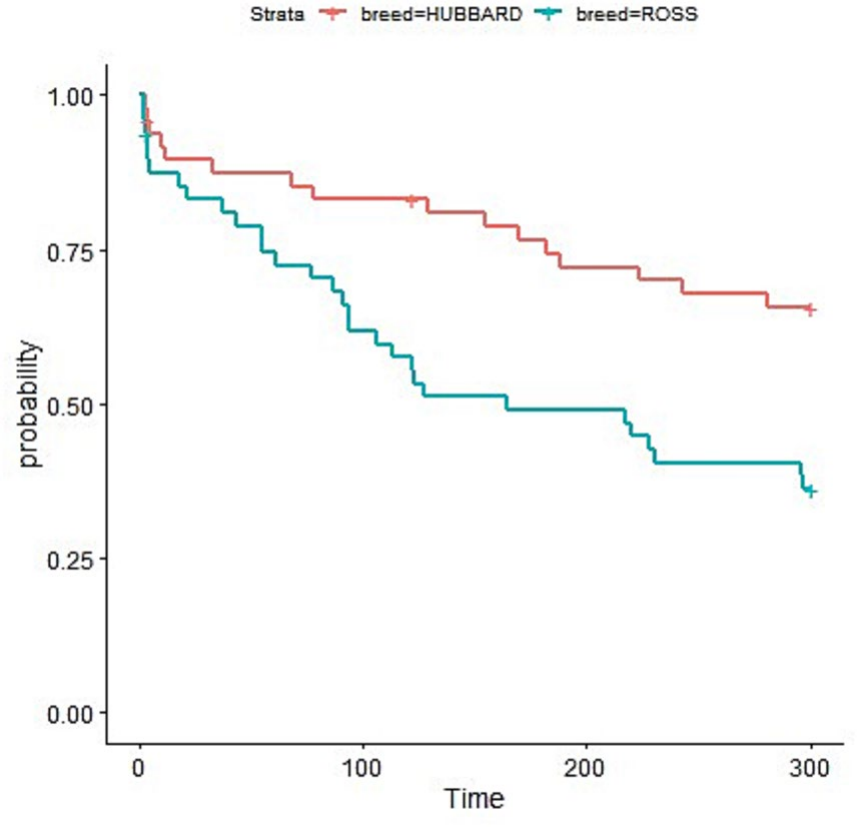
Latency to first attempt to stand (measured in seconds) during the tonic immobility test performed at slaughter age (target BW ≈ 2.5 kg) in both Ross 308 and Hubbard JA757 chickens.

Broiler breed had a significant effect on indicators of fear assessed during the novel environment test (Table 4). Ross 308 chickens exhibited a shorter latency and a higher number of vocalizations than Hubbard JA757 chickens (*p* < 0.05). Early-fed broiler chickens vocalized less compared to not early-fed chickens (Δ = −27.8 on average; *p* < 0.01). During the tonic immobility test, Hubbard JA757 exhibited a shorter latency to stand than Ross 308 chickens (Figure 4).

**Table 4:** Effects of broiler breed (Ross 308 and Hubbard JA 757) and early feeding (EF; YES or NO) on behaviors measured during the novel environment test performed on day 6.

	Treatments^1^					*p*-values		
	R + EF	R-EF	H + EF	H-EF	Pooled SD^2^	Broiler breed	EF	Broiler breed×EF
Latency to first vocalization (sec)	5.3	4.0	14.6	8.1	2.0	<0.01	0.40	0.74
Latency to first escape attempt (sec)	45.5	57.0	66.2	79.3	12.2	0.05	0.14	0.75
No. of vocalizations	134.7	167.4	104.4	127.3	10.6	<0.01	0.02	0.66
No. escape attempts	0.5	1.0	0.4	0.5	0.2	0.70	0.24	0.77

1 R + EF = Ross 308 chickens with early feeding; R-EF = Ross 308 chickens without early feeding; H + EF=Hubbard JA757 chickens with early feeding; H-EF = Hubbard JA757 chickens without early feeding.

2 SD, standard deviation.

## 4 Discussion

The main aim of the current study was to investigate the effects of both breed and EF and their interaction on jejunum microbiota, immune development, and behavior at two different target BW representing early life and slaughter age (approximately 200 g and 2.5 kg). As shown by the results, in the early phase of broiler life (target BW ≈ 200 g), EF had a significant effect on fear response and cytokine expression in the jejunum. However, EF did not produce long-term effects, as there were no observed effects on jejunal microbiota, cytokine expression, and fearfulness at ≈2.5 kg target BW in either breed. This is in agreement with previous studies, indicating that EF is an important nutritional strategy that is beneficial for broiler chickens, particularly in the first phase of their life (4, 7, 9). De Jong et al. (22), da Silva et al. (32), and Marcato et al. (9) showed that provision of feed and water immediately post-hatch can have a positive effect on body weight gain, ADFI, and FCR in the starter phase of broilers, whereas early post-hatch fasting for 24 h can be detrimental to the starter phase weight gain and performance of broilers (33). In addition to performance, EF can affect gut health by accelerating yolk absorption and stimulating intestinal health, growth, and absorption via enhancement of villus height and crypt depth in the duodenum and jejunum (7, 34). Moreover, it is known that EF can affect the gut microbiota (7), levels of the proinflammatory cytokines IL-1β, IL-12p40, IFN-*γ*, and the anti-inflammatory cytokine IL-10 (35), and behavior (23) in broiler chickens. Li et al. (7) reported a higher microbial richness and a lower relative abundance of *Proteobacteria* and *Escherichia* in the starter phase (day 21) in chickens receiving EF compared to chickens with 48 h delayed nutrition. A higher microbial richness could provide a species-rich microecosystem to withstand external stressors more effectively and maintain the healthy development of the intestinal tract (36, 37), whereas a lower percentage of *Proteobacteria* and *Escherichia* indicates a healthy intestinal environment and could contribute to higher performance of broilers (7). In the current study, EF did not affect the jejunal microbiota at either target BW of 200 g or 2.5 kg, which agrees with the results of a similar study analyzing fecal samples (9). Possibly, in the present experiment, dietary and environmental effects overshadowed the effects of early feeding as it has been shown that these can have significant effects on intestinal microbiota composition (38). However, it could still be that direct microbiota changes do occur and that perpetual changes are being observed, for example, significant (short term) changes in the cytokine mRNA levels in the jejunum of broilers at an early time point (target BW ≈ 200 g). Particularly, broiler chickens without EF and from the fast-growing Ross 308 line were most affected, showing higher expression of IFNγ as compared to the other treatments. Interferon-gamma in poultry is a proinflammatory cytokine that is involved in the host defense against infection (39, 40). High levels of this cytokine can contribute to an increase in intestinal permeability by modifying tight junction distribution within the intestinal tract (3, 41). Other cytokines, such as IL-17 or IL-22, are critical for the maintenance of mucosal homeostasis and protect the epithelial integrity (42) by downregulating the action of proinflammatory cytokines (10). Thus, higher mRNA levels of IFNγ in Ross 308 without EF may indicate that these broilers are more challenged than the rest. Moreover, the selection for genetic traits, such as rapid growth and feed intake, in Ross 308 chickens might have resulted in a higher sensitivity to EF compared to Hubbard JA757. As a consequence, this nutritional strategy might be beneficial, especially for faster-growing breeds, such as Ross 308, which can have a different development and thus a different immune status as compared to slower-growing breeds.

Early access to water and feed has been shown to act as an early life environmental enrichment, thus stimulating brain and cognitive development and the ability to express early fear responses in chicks (23, 43). Previous studies reported that a lower number of vocalizations and escape attempts in a novel environment are signs of reduced fearfulness in broiler chickens (30, 44, 45). This is in agreement with the current results showing that early-fed broiler chickens vocalized less compared to not early-fed chickens during the NE test. Most likely, the early exposure to feed and water reduced the fear responses of the animals when placed in social isolation in a novel environment. Moreover, the lower number of vocalizations recorded in early-fed chicks could also be interpreted as a positive effect of enrichment provided by both feed and water at the hatchery or/and reduced hunger of chicks in the first days post-hatch.

In addition to the effects of EF, this is one of the first studies (9) to investigate the effects of breed in combination with EF on jejunal microbiota, cytokine expression, and behavior. Although we did not observe other interactions in addition to breed and EF on cytokine levels, breed was revealed to be an important factor shaping the jejunal microbiota, fear behavior at both early and late time points, and immune cells at slaughter age. In contrast to previous studies showing no effects of these breeds on the microbiota (9, 46, 47), Hubbard JA757 had a higher microbial richness at the late time point (target BW ≈ 2.5 kg) and the results of PCoA showed that there were two different clusters of microbiota at both timepoints. Moreover, additional analysis at the genus level showed significant differences between breeds at a target BW ≈ 2.5 kg. The differences might be due to the use of jejunum samples, compared to fecal swabs used in previous studies. Small intestinal samples can be more representative and show more profound effects on both alpha and beta diversity (48, 49). The higher microbial richness in Hubbard JA757 chickens at slaughter age might have important implications for the poultry sector with regard to this slower-grower breed. A related study (9) found that, at slaughter age, this breed produced significantly less endotoxins in the feces compared to Ross 308 chickens but failed to prove differences in fecal microbiota. The results of the current study might therefore be the explanation for the differences in endotoxin release, which are a health concern for both animals and humans (50, 51). However, it should also be mentioned that the differences observed in both intestinal microbiota (in the current study) and endotoxins (in the previous study (9)) might be also attributed to age differences (34 vs. 50 days) between the two breeds at the late sampling moment, which is a known factor to affect both gut microbiota and endotoxins (6, 52). The difference in age at the late time point might also be the main factor responsible for differences in the number of immune cells in the blood among the two breeds, as reported previously (53). In this context, it is difficult to know the exact cause of the increased activation of the immune system, and it is not clear whether this has positive or negative implications. Thus, a better understanding of microbiota composition, immune development, and their interplay in these two breeds at similar ages and BW is needed.

Breed affected behavior as well at both time points. Ross 308 chickens were more fearful than Hubbard JA757 during the NE test, as indicated by a shorter latency to the first vocalization and a higher number of vocalizations. This might be explained by the genetic selection for faster growth in the Ross 308, which is known to have negative consequences on different traits, such as heat tolerance, locomotion, body condition, and fear behavior (54–57). During the TI test at a late time point, Hubbard JA757 chickens exhibited a shorter latency to stand than Ross 308 chickens. This suggests that Hubbard JA757 were still less fearful in the long term, as a longer latency to stand after TI and a longer duration of TI have been associated with higher levels of fearfulness in broiler chickens at both early (1–4 days old) and late (30–35 days old) ages (23, 30, 58). Fear can be a welfare problem and can have an adverse impact on the productivity of broiler chickens (56); thus, reducing the level of fearfulness is important for the poultry sector.

## 5 Conclusion

This study showed that both EF and breed are important factors that impact different intestinal health and behavioral measures in broiler chickens. EF primarily influenced the early production phase, while breed had a significant impact on gut microbiota, immune development, and behavior, extending to slaughter weight. EF contributed to reduced fear responses in chicks. Slower-grower Hubbard JA757 exhibited a higher gut microbial richness at slaughter age and less fearfulness at both the beginning and the end of the production period compared to faster-growing Ross 308 chickens. Moreover, the effects of the interaction between EF and breed were present on cytokine expression at an early age. Ross 308 without EF exhibited the highest expression levels of IFNγ, which might be indicative of a divergence in immune development. The use of EF can be helpful, especially for the fast-growing chicks during the first days of their life but without long-term effects on key health and welfare indicators. Production of a slower-growing breed might be the new focus for the poultry sector given some beneficial effects on gut microbiota and fear responses. Overall, these factors should be also tested outside experimental/controlled conditions to observe whether a commercial set-up can support these findings.

## Data Availability

The data presented in the study are deposited in the NCBI bioproject for the 16S sequencing data repository, accession number PRJNA975731.

## Supplementary material

SUPPLEMENTARY FIGURE S1 Microbiota composition on genus level (top 10 most abundant genera) of jejunum samples collected from broiler chickens at a target body weight (BW)≈200g (Figure 1A) and at a target BW≈2.5kg (Figure 1B). The figure shows the comparison between the breed (Ross 308 vs. Hubbard JA757) and early feeding (yes vs.no).
