# Supplementary figures and images for: Effects of breed and early feeding on intestinal microbiota, inflammation markers, and behavior of broiler chickens
Source: Front Vet Sci. 2024 Dec 2;11:1492274. doi: 10.3389/fvets.2024.1492274 (PMC11648218; doi:10.3389/fvets.2024.1492274)

A

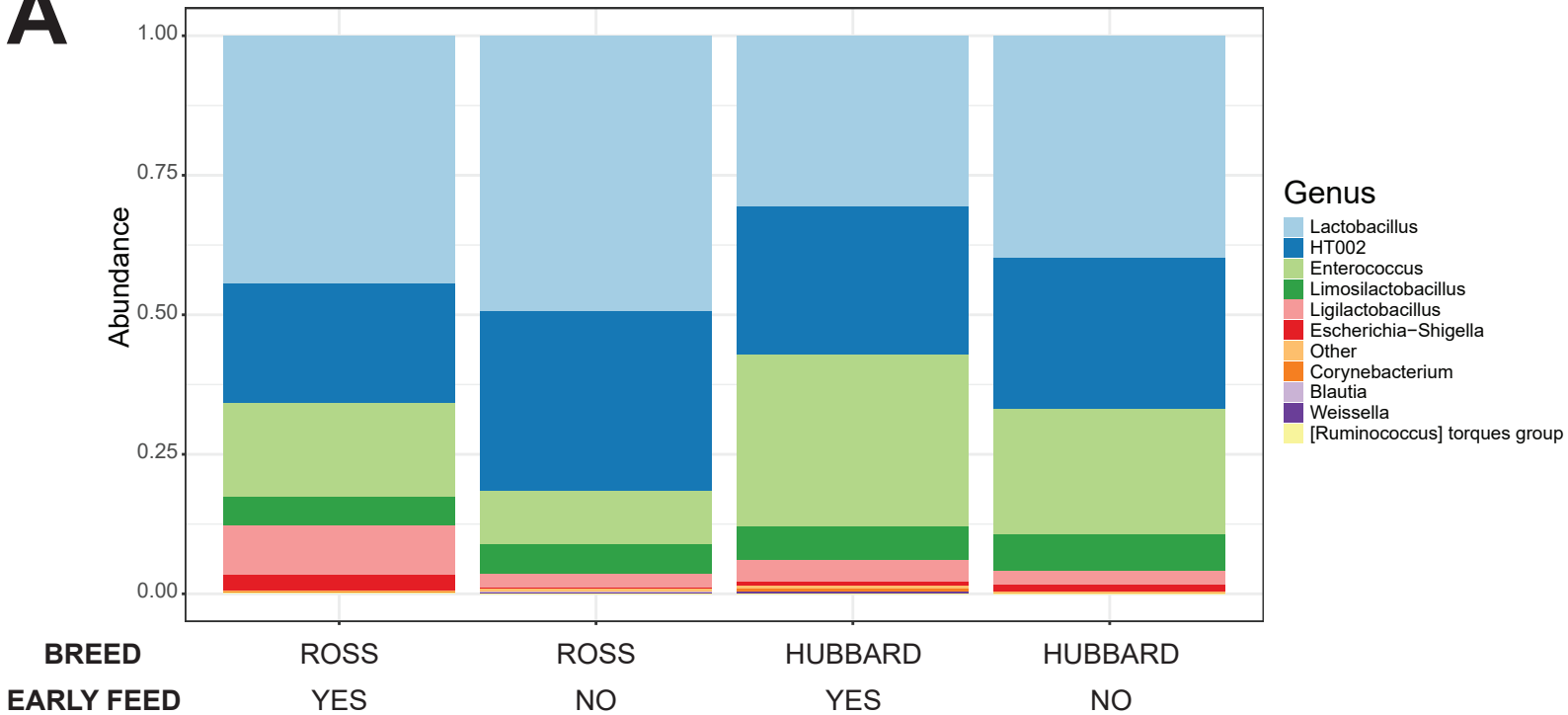

B

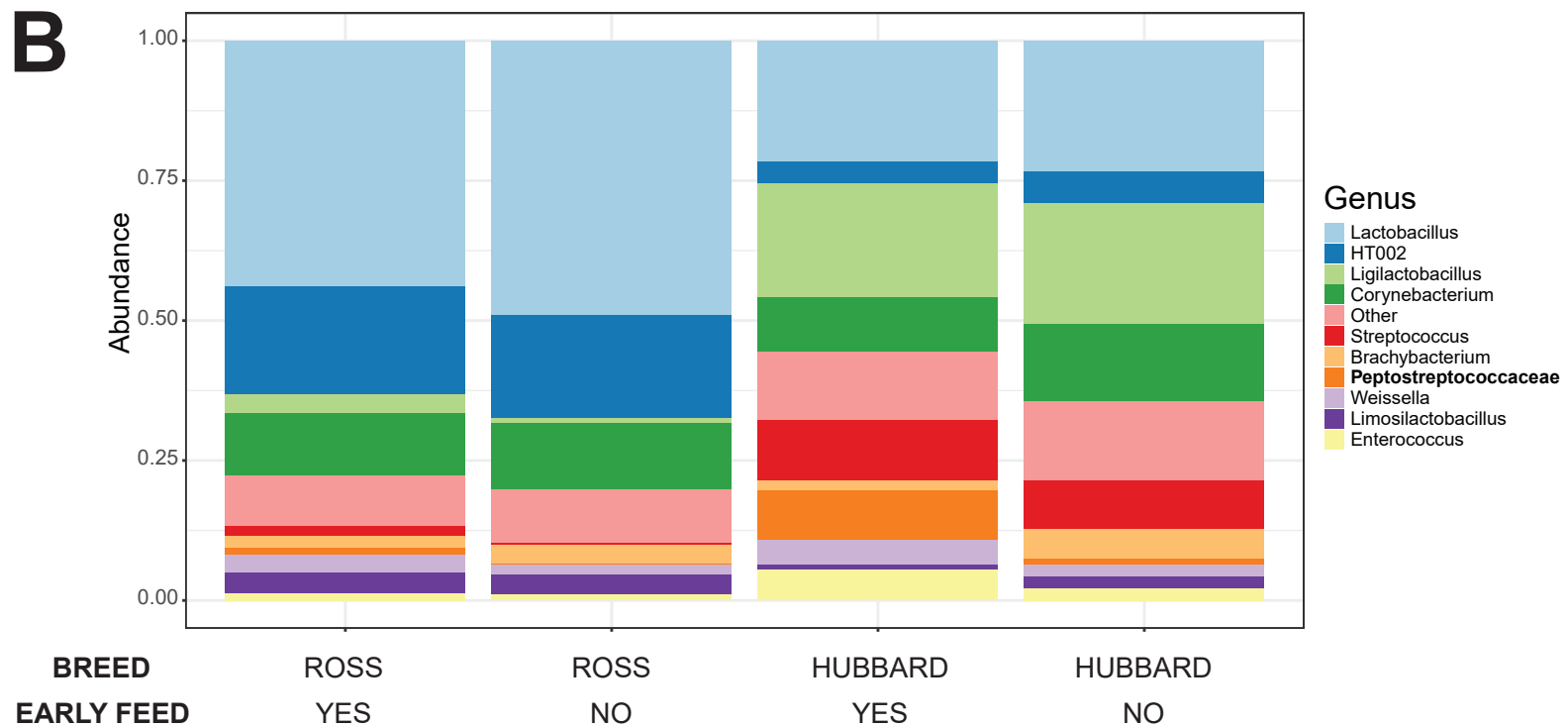

Supplement: SUPPLEMENTARY FIGURE S1 — Microbiota composition on genus level (top 10 most abundant genera) of jejunum samples collected from broiler chickens at a target body weight (BW)≈200g (Figure 1A) and at a target BW≈2.5kg (Figure 1B). The figure shows the comparison between the breed (Ross 308 vs. Hubbard JA757) and early feeding (yes vs.no). [file Image_1.pdf]
