# Supplementary material for: Effects of breed and early feeding on intestinal microbiota, inflammation markers, and behavior of broiler chickens
Source: Front Vet Sci. 2024 Dec 2;11:1492274. doi: 10.3389/fvets.2024.1492274 (PMC11648218; doi:10.3389/fvets.2024.1492274)
Supplement: Supplementary file 2 [file Table_1.docx]

**Supplementary Table 1.** Primer sequences used for real-time quantitative PCR.

| Gene | Forward Primer | Reverse Primer |
| --- | --- | --- |
| IFNγ | GTGAAGAAGGTGAAAGATATCATGGA | GCTTTGCGCTGGATTCTC A |
| IL-17 | TATCAGCAAACGCTCACT GG | AGTTCACGCACCTGGAATG |
| IL-22 | CAGACTCATCGGTCAGCAAA | GGTACCTCTCCTTGGCCTCT |
| PPIA | CCATTTACGGGGAGAAGTTT | CAGTGCAGATGAAGAACTGG |
| ACTB | GCCCTGGCACCTAGCACAAT | GCGGTGGACAATGGAGGGT |
