# Supplementary material for: Effects of breed and early feeding on intestinal microbiota, inflammation markers, and behavior of broiler chickens
Source: Front Vet Sci. 2024 Dec 2;11:1492274. doi: 10.3389/fvets.2024.1492274 (PMC11648218; doi:10.3389/fvets.2024.1492274)
Supplement: Supplementary file 3 [file Table_2.docx]

**Supplementary Table 2.** Significant log2fold changes in top-10 genera at approximately 2.5 kg body weight for Hubbard JA757 versus Ross 308 broiler chickens, within the early fed and non-early fed treatment groups, and the adjusted p-values for comparison between the breeds within the non-early fed and early fed groups.

|  | **Log2Fold change** | **p-adjusted** |
| --- | --- | --- |
| **Hubbard-EF versus Ross-EF** |  |  |
| *Ligilactobacillus* | 5.09 | <0.001 |
| HT—2 | -3.45 | <0.001 |
| *Streptococcus* | 5.70 | <0.001 |
| *Enterococcus* | 1.39 | 0.012 |
| *Lactobacillus* | -1.36 | 0.012 |
| *Limosilactobacillus* | -1.69 | 0.012 |
| **Hubbard+EF versus Ross+EF** |  |  |
| *Peptostreptococcaceae*^1^ | 5.41 | <0.001 |
| *Enterococcus* | 2.43 | <0.001 |
| *Ligilobacillus* | 3.57 | <0.001 |
| HT002 | -2.59 | <0.001 |
| *Limosilactobacillus* | -2.11 | <0.001 |

^1^ Phylum
